# Do visual analogue scale (VAS) derived standard gamble (SG) utilities agree with Health Utilities Index utilities? A comparison of patient and community preferences for health status in rheumatoid arthritis patients

**DOI:** 10.1186/1477-7525-4-25

**Published:** 2006-04-20

**Authors:** Amir Adel Rashidi, Aslam H Anis, Carlo A Marra

**Affiliations:** 1 Centre for Clinical Epidemiology and Evaluation, Faculty of Pharmaceutical Sciences, University of British Columbia, Canada; 2 MHA Program, Department of Health Care and Epidemiology, Faculty of Medicine, University of British Columbia, Canada; 3 Faculty of Pharmaceutical Sciences, University of British Columbia, Vancouver, BC, Canada; 4 Centre for Clinical Epidemiology and Evaluation, Vancouver Coastal Health Research Institute, Vancouver, BC, Canada

## Abstract

**Background:**

Assessment of Health Related Quality of Life (HRQL) has become increasingly important and various direct and indirect methods and instruments have been devised to measure it. In direct methods such as Visual Analog Scale (VAS) and Standard Gamble (SG), respondent both assesses and values health states therefore the final score reflects patient's preferences. In indirect methods such as multi-attribute health status classification systems, the patient provides the assessment of a health state and then a multi-attribute utility function is used for evaluation of the health state. Because these functions have been estimated using valuations of general population, the final score reflects community's preferences. The objective of this study is to assess the agreement between community preferences derived from the Health Utilities Index Mark 2 (HUI2) and Mark 3 (HUI3) systems, and patient preferences.

**Methods:**

Visual analog scale (VAS) and HUI scores were obtained from a sample of 320 rheumatoid arthritis patients. VAS scores were adjusted for end-aversion bias and transformed to standard gamble (SG) utility scores using 8 different power conversion formulas reported in other studies. Individual level agreement between SG utilities and HUI2 and HUI3 utilities was assessed using the intraclass correlation coefficient (ICC). Group level agreement was assessed by comparing group means using the paired t-test.

**Results:**

After examining all 8 different SG estimates, the ICC (95% confidence interval) between SG and HUI2 utilities ranged from 0.45 (0.36 to 0.54) to 0.55 (0.47 to 0.62). The ICC between SG and HUI3 utilities ranged from 0.45 (0.35 to 0.53) to 0.57 (0.49 to 0.64). The mean differences between SG and HUI2 utilities ranged from 0.10 (0.08 to 0.12) to 0.22 (0.20 to 0.24). The mean differences between SG and HUI3 utilities ranged from 0.18 (0.16 to 0.2) to 0.28 (0.26 to 0.3).

**Conclusion:**

At the individual level, patient and community preferences show moderate to strong agreement, but at the group level they have clinically important and statistically significant differences. Using different sources of preference might alter clinical and policy decisions that are based on methods that incorporate HRQL assessment. VAS-derived utility scores are not good substitutes for HUI scores.

## Background

In recent years, cost-utility analysis has emerged as a common methodology for the economic evaluation of health care strategies. This approach makes use of quality adjusted life years (QALYs) to assess the effectiveness of health care interventions. Neumann et al. stated that "QALYs represent the benefit of a health intervention in terms of time in a series of quality-weighted health states" in which the quality weights reflect the desirability of living in the state [1]. Therefore, once the quality weights are obtained for each health state experienced by an individual, they are multiplied by the duration of time spent in the health state. The products of these calculations are then summed to obtain the total number of QALYs.

Preference-based assessments, which can be categorized into direct and indirect measures, are often used to obtain the desirability or preferences for health states. In direct measures, the respondent directly "assesses" and "evaluates" a health state on a scale of 0.00 (death) to 1.00 (perfect health). The health states that are evaluated in the direct approach can be hypothetical or can be the respondent's own subjectively defined current health state (SDCS) [2].

In indirect measures, the respondent provides information regarding their health status by completing a multi-attribute health status classification system questionnaire such as the Health Utilities Index Mark 2 (HUI2) [3] and Mark 3 (HUI3) [4], the Quality of Well Being (QWB) [5], the EuroQol (EQ-5D) [6,7] and the Short-Form 6-D (SF-6D) [8]. The "valuation" of that assessment then comes from a scoring formula which is typically based on preferences for health states from a general population sample.

Direct methods include the visual analog scale (VAS), and standard gamble (SG) techniques. The SG requires respondent's concentration, sound cognitive functioning, and requires experienced interviewers with effective props [9,10]. Since multi-attribute health status classification system questionnaires can be self-administered, or completed through telephone interviews, they have been more widely used.

Alternatively, some researchers have tried to use simple indirect techniques such as the VAS and then converted the scores to SG utilities using power transformations [11,12].

Although different variations of VAS have been frequently used as a simple method of preference measurement, recently some concerns regarding their validity have been raised [13-15]. For example, the VAS anchors are often not well defined and several measurement biases such as context bias and end-aversion bias may occur. However, there is evidence that limited and cautious use of the VAS is useful and appropriate [16].

Different approaches, considering preferences of different population subgroups, have been used to elicit the "values" of various health states [17]. However, the two main sources of values are individual patients and the general population. On one hand, it is felt that patients who have directly experienced a health state can better assess its effect on their HRQL and express a true preference. On the other hand, members of the general public are less likely to have self-interest or strategic bias in their evaluations and thus may be more objective. Moreover, since the general public incurs the cost of resource allocation decisions, it may be more reasonable to measure preferences for health states and benefits from the general public's perspective [17].

Currently, economic evaluation guidelines recommend using preference-based valuation methods in which the general public is the source of values [18,19]. However, it is not clear whether community members value a given health state the same as patients who are experiencing that health state. If there are significant differences between these, then the results of economic evaluations could change depending on the preference source. Although several studies have shown that patient-based and community-based utilities are significantly different [10,20-22], some other studies have shown otherwise [23,24]. Recently, Feeny and colleagues reported differences between utilities derived from the HUI2 and SG at the individual level, but at the same time observed no difference at the group level [2,25].

As such, our objective was to assess the agreement between indirectly obtained community preferences and directly obtained patient preferences in a sample of rheumatoid arthritis patients.

## Methods

### Study sample

A sample of patients with a rheumatologist-confirmed diagnosis of rheumatoid arthritis (RA) was previously assembled for a longitudinal study to examine the reliability and responsiveness of the indirect utility instruments [26-28]. All participants provided informed consent and ethical approval for this study was obtained through the University of British Columbia's Behavioural Ethics Committee. Three hundred and twenty patients took part in the study and data were gathered at three intervals: baseline (Assessment A), after 3 months (Assessment B) and after 6 months (Assessment C).

### Indirect and direct assessment of preferences for health states

The study questionnaire included the HUI Mark 2 and 3, and the EQ-5D. Patients' preferences for their current health state were obtained using a VAS as part of the EQ-5D questionnaire. The EQ-5D questionnaire [6,7] consists of a descriptive health profile including five domains and a health thermometer (VAS) which represents a subjective, global evaluation of the respondent's health status on a vertical scale between 0 and 100, where 0 (the bottom anchor) represents the worst imaginable health state and 100 (the top anchor) represents the best imaginable health state.

### Adjustment for end-aversion bias

Many respondents are unwilling to place health states at the extreme portions of a continuous scale, leading to end-aversion bias [29,30]. The magnitude of end-aversion bias in VAS has been investigated using the pair-wise comparison method [16,31]. It was found that, on average, health states close to the healthy end are placed 1.78 times too far away, whereas at the unhealthy end, there is minimal bias. As such, only VAS scores placed in the upper quarter of the scale were adjusted and, in order to maintain the relative position of other scores, a positive linear transformation was performed. No adjustment was performed for the unhealthy end (closer to zero). This procedure is similar to the adjustment method performed in development of HUI3 [4].

### Transformation of VAS scores to utility scores

Utilities for the respondent's SDCS were derived using a transformation function to convert adjusted VAS values (V) to SG utility scores (U). After adjustment for end-aversion bias, VAS scores first were transformed from a 0–100 scale to a 0.00–1.00 scale. Then, power functions were used to transform the data to SG utility scores. Power conversion is the most common transformation function used for mapping the relationship between VAS scores and SG utilities [16]. All eight different functions, previously described by Torrance [16], were used to perform the transformations (Table 1).

**Table 1:** Different power functions reported for transforming VAS values (V) to SG utilities (U)*

**Function number**	**Equation**	**Reference**
1	U = 1-(1-V)^1.6^	Torrance et al.[51]
2	U = 1-(1-V)^2.2^	Wolfson et al.[52]
3	U = 1-(1-V)^2.3^	Torrance et al.[3]
4	U = 1-(1-V)^2.4^	Feeny et al.[53]
5	U = 1-(1-V)^2.7^	Krabbe et al.[54]
6	U = 1-(1-V)^2.9^	Feeny et al.[53]
7	U = V^0.56^	Furlong et al.[55]
8	U = V^0.47^	Furlong et al.[55] and Le Gales et al.[56]

*Obtained from Torrance [16]

### HUI2 and HUI3

Each HUI system includes a health status classification system and a multi-attribute utility scoring formula. The HUI2 consists of questions regarding seven dimensions of health status: sensation, mobility, emotion, cognition, self-care, pain, and fertility. Because each question describes 3 to 5 levels of a health attribute, the HUI2 can describe a total of 24,000 unique health states [3]. The HUI3 consists of questions regarding eight dimensions of health status: vision, hearing, speech, ambulation, dexterity, emotion, cognition, and pain. Because each question describes 5 to 6 levels of a health attribute, the HUI3 can describe a total of 972,000 unique health states [4]. The multi-attribute utility scoring formula calculates a utility score that reflects community preferences for the respondent's assessment of his or her health status. The scoring formulae are based on SG utilities derived mainly from power conversions of VAS scores. The overall utility scores obtained from HUI2 range from -0.03 to 1.0 and for HUI3 from -0.36 to 1.0, where 1.0 represents a HRQL of perfect health and 0 represents a HRQL of death. However, the overall utility scores for HUI 2 and HUI3 can also be calculated such that 0.00 represents the worst imaginable health state and 1.00 represents the perfect health [3,4].

### Statistical analysis

The HUI2 and HUI3 scores were considered indirect community-preference-based utility scores. VAS scores were adjusted for end-aversion bias, and after conversion to SG utility scores were considered direct patient-preference-based utility scores (adjusted SG utility). SG utility scores were also calculated without adjusting for end-aversion bias (unadjusted SG utility). Both adjusted and unadjusted SG utility scores were calculated using each of the eight power conversion formulae (Table 1).

VAS values (and therefore the obtained SG utility scores) are bound between 0.00 and 1.00. In order to avoid comparing agreement between two utility measures with dissimilar ranges, the HUI2 and HUI3 scores were calculated in a 0.00 to 1.00 scale in this study.

Descriptive statistics are presented for each set of utility scores. Agreement between SG utility scores and HUI2 and HUI3 scores, at the individual level, was assessed using the Pearson Correlation Coefficient and the Intraclass Correlation Coefficient (ICC) with a two-way mixed effect model such that the respondent effect was random and the measure effect was fixed [32]. Both the adjusted and unadjusted SG utility scores were examined separately. Interpretation of the strength of agreement using ICC scores was taken from the framework proposed by Guyatt et al. (strong: ICC>0.50; moderate: ICC = 0.35–0.50; weak: ICC = 0.20–0.34; negligible: ICC = 0.00–0.19) [33]. Paired sample t-tests were used to assess agreement between direct and indirect utility scores at the group level. All the above tests were performed to assess agreement between the HUI scores and each SG utility score calculated from the different power conversions (8 adjusted and 8 unadjusted). The minimal important difference (MID) of utilities was considered to be 0.03 [9].

A 0.05 level of significance was used in all analyses. ICC analyses were carried out using SPSS version 11.5. All other statistical analyses were performed using SAS version 8.2.

## Results

### Respondents

From the 320 participants who received the baseline questionnaire (Assessment A), 308 completed the VAS scores as part of EQ-5D questionnaire, and 307 and 306 global utility scores could be generated using HUI scoring functions for the HUI2 and HUI3, respectively. Of these, 303 respondents had both VAS and HUI2 scores and 302 had both VAS and HUI3 scores. Summary statistics for the eight different SG scores derived from VASs and HUI2 and HUI3 scores are presented in Table 2. More information regarding the demographic characteristics and disease severity of the study population has been published elsewhere [27,28].

**Table 2:** Summary statistics for HUI2, HUI3 and SG utilities obtained from transformation of VAS scores by different power conversions

**Assessment A**		**N**	**Mean**	**SD**	**Median**	**Min.**	**Max.**
**Unadjusted**	**SG1**^**1**^	308	0.79	0.18	0.85	0.08	1.00
	**SG2**	308	0.86	0.16	0.93	0.11	1.00
	**SG3**	308	0.87	0.15	0.93	0.11	1.00
	**SG4**	308	0.88	0.15	0.94	0.12	1.00
	**SG5**	308	0.90	0.14	0.96	0.13	1.00
	**SG6**	308	0.91	0.13	0.97	0.14	1.00
	**SG7**	308	0.77	0.15	0.81	0.18	0.99
	**SG8**	308	0.80	0.13	0.84	0.24	1.00
							
**Adjusted**	**SG1**	308	0.84	0.18	0.92	0.09	1.00
	**SG2**	308	0.90	0.15	0.97	0.12	1.00
	**SG3**	308	0.91	0.14	0.97	0.13	1.00
	**SG4**	308	0.91	0.14	0.98	0.13	1.00
	**SG5**	308	0.93	0.13	0.99	0.15	1.00
	**SG6**	308	0.93	0.12	0.99	0.16	1.00
	**SG7**	308	0.82	0.15	0.88	0.19	1.00
	**SG8**	308	0.85	0.13	0.90	0.26	1.00
							
	**HUI2**	307	0.72	0.19	0.75	0.12	1.00
	**HUI3**	306	0.66	0.21	0.68	0.14	1.00

^1^Numbers indicate the power conversions (listed in Table 1) used to transform VAS scores to SG scores.

### Individual level agreement between direct and indirect utilities

Individual level ICCs and Pearson correlation coefficients were calculated where all 3 scores (VAS, HUI2 and HUI3) were available. The complete ICC analysis of Assessment A along with the Pearson correlation coefficients is presented in Table 3. In general, based on ICC results, moderate to strong agreement was found between SG utilities and HUI2 and HUI3 utilities at the individual level.

**Table 3:** Pearson (r) and Intraclass (ICC) correlation coefficients between eight different SG scores (both adjusted and unadjusted) and HUI2 and HUI3. The 95% confidence intervals for ICCs are included

**SG utility**		**HUI2**			**HUI3**		
**Assessment A**		**r**	**ICC**	**95% CI**	**r**	**ICC**	**95% CI**
**Unadjusted**	**SG1**	60%	0.57	0.49 to 0.64	60%	0.60	0.52 to 0.66
	**SG2**	55%	0.54	0.46 to 0.62	58%	0.56	0.47 to 0.63
	**SG3**	55%	0.54	0.45 to 0.61	58%	0.55	0.47 to 0.62
	**SG4**	55%	0.53	0.45 to 0.61	57%	0.54	0.46 to 0.62
	**SG5**	54%	0.51	0.42 to 0.59	56%	0.52	0.43 to 0.60
	**SG6**	53%	0.50	0.41 to 0.58	55%	0.50	0.41 to 0.58
	**SG7**	58%	0.60	0.48 to 0.63	62%	0.58	0.49 to 0.68
	**SG8**	58%	0.53	0.45 to 0.61	61%	0.54	0.46 to 0.62
							
**Adjusted**	**SG1**	55%	0.55	0.47 to 0.62	58%	0.57	0.49 to 0.64
	**SG2**	53%	0.51	0.42 to 0.59	55%	0.52	0.43 to 0.60
	**SG3**	53%	0.51	0.42 to 0.58	55%	0.51	0.42 to 0.59
	**SG4**	52%	0.50	0.41 to 0.58	55%	0.50	0.41 to 0.58
	**SG5**	51%	0.47	0.38 to 0.56	53%	0.47	0.38 to 0.55
	**SG6**	50%	0.45	0.36 to 0.54	52%	0.45	0.35 to 0.53
	**SG7**	57%	0.55	0.47 to 0.62	60%	0.56	0.48 to 0.64
	**SG8**	57%	0.53	0.44 to 0.60	60%	0.53	0.44 to 0.61

The ICCs (95% confidence interval) between the adjusted SG and HUI2 utilities in Assessment A ranged from 0.45 (0.36 to 0.54) to 0.55 (0.47 to 0.62), where most ICCs were more than 0.50. ICCs between the unadjusted SG and HUI2 utilities were all higher than the ICCs between the corresponding adjusted SG and HUI2 utilities with no ICC below 0.50. These results show that agreement between the SG and HUI2 scores at the individual level is strong. However, there is only moderate agreement at the individual level between the SG and HUI3 utilities. The ICC (95% confidence interval) between the adjusted SG and HUI3 utilities in Assessment A ranged from 0.45 (0.35 to 0.53) to 0.57 (0.49 to 0.64). ICCs between the unadjusted SG and HUI3 utilities were all higher than the ICCs between the corresponding adjusted SG and HUI3 utilities. In almost all measurements, the Pearson correlation coefficients slightly exceeded the corresponding ICCs. However, none of the differences were statistically significant. The analyses of Assessments B and C completely support these findings (data not shown).

### Group level agreement between direct and indirect utilities

Results of the comparison between the mean SG utilities, HUI2, and HUI3 scores using paired sample t-tests are reported in Table 4. The differences between the SG utilities and the HUI scores (the HUI score was subtracted from the SG utility) were calculated for every respondent and then the mean of the differences was examined for statistical significance and clinical importance.

**Table 4:** Results of the comparison between mean SG utilities and HUI2 and HUI3 scores using paired sample t-tests

**SG utility**		**HUI2**			**HUI3**		
Assessment A		N	Mean Difference	95% CI	N	Mean Difference	95% CI
**Unadjusted**	**SG1**	303	0.07	0.05 to 0.09	302	0.13	0.11 to 0.15
	**SG2**	303	0.14	0.12 to 0.16	302	0.20	0.18 to 0.22
	**SG3**	303	0.15	0.13 to 0.17	302	0.21	0.19 to 0.23
	**SG4**	303	0.16	0.14 to 0.18	302	0.22	0.20 to 0.24
	**SG5**	303	0.18	0.16 to 0.20	302	0.24	0.22 to 0.26
	**SG6**	303	0.19	0.17 to 0.21	302	0.25	0.23 to 0.27
	**SG7**	303	0.05	0.03 to 0.07	302	0.11	0.09 to 0.13
	**SG8**	303	0.09	0.07 to 0.11	302	0.15	0.13 to 0.17
							
**Adjusted**	**SG1**	303	0.12	0.11 to 0.14	302	0.18	0.16 to 0.20
	**SG2**	303	0.18	0.16 to 0.20	302	0.24	0.22 to 0.26
	**SG3**	303	0.19	0.17 to 0.21	302	0.25	0.23 to 0.27
	**SG4**	303	0.19	0.18 to 0.21	302	0.25	0.23 to 0.27
	**SG5**	303	0.21	0.19 to 0.23	302	0.27	0.25 to 0.29
	**SG6**	303	0.22	0.20 to 0.24	302	0.28	0.26 to 0.30
	**SG7**	303	0.10	0.08 to 0.12	302	0.16	0.14 to 0.18
	**SG8**	303	0.13	0.11 to 0.15	302	0.19	0.17 to 0.21

In general, the mean differences between the SG utilities and HUI2 and HUI3 scores were important and statistically significant. They were all positive, showing that the SG utilities consistently exceeded HUI utilities. The mean differences between adjusted SG utilities and HUI2 scores were considerable but not so large. The mean (95% confidence interval) ranged from 0.10 (0.08 to 0.12) to 0.22 (0.20 to 0.24). The mean differences between the adjusted SG utilities and HUI3 scores were larger, ranging from 0.18 (0.16 to 0.20) to 0.28 (0.26 to 0.30).

As expected, the mean differences between the unadjusted SG utilities and HUI2 scores were all smaller than the mean differences between the corresponding adjusted SG utilities and HUI2 scores, but all were important and statistically significant. The same was true for HUI3 scores. Analysis of Assessments B and C showed the same results (data not shown).

## Discussion

Our results indicate that at the individual level, good agreement exists between SG and HUI utility scores. The agreement between SG and both HUI2 and HUI3 utilities is generally strong (ICC>0.50). Also, at the group level we found that SG and HUI utilities have important and significant differences. The differences were relatively large and systematically in the same direction. Interestingly, our findings are in contrast with the results from Feeny et al. [2,25] and others [21,34,35].

### Agreement between direct and indirect utilities at the individual level

Why is agreement less than perfect? How can we explain the approximately 50 percent disagreement between direct and indirect utilities? And what are the possible sources of disagreement between these utilities?

The first explanation could be that direct and indirect utilities measure preferences for health states from different perspectives. While SG and HUI scores are both utilities, in direct measurement (SG), patient preferences are the basis of the health status valuation, whereas in indirect assessment (HUI), the valuation is based on community preferences. In the direct SG measurement of a patient's current health state, the patient makes a subjective assessment of his or her health status and then gives his or her personal evaluation of that health state. However, in multi-attribute health status classification systems, such as the HUI2 and HUI3, the patient provides the assessment of his or her health state and then a multi-attribute utility function (which has been estimated using the preferences of general population) is used to evaluate the health state [25].

This difference in perspective might lead to unequal results for utility measurements which can be explained by a phenomenon called response shift. Response shift occurs when the meaning of one's self-evaluation changes [36]. In general, patients who have experienced a chronic health condition, such as RA, may give that health state a higher value compared to the general public. Healthy individuals might have an exaggerated fear of the morbidity and disability associated with such a chronic illnesses, while chronically ill patients often learn how to cope with their condition over time. Specifically, studies of rheumatic diseases have shown that patients' self-reported functional limitation and their actual physical impairment are considerably different [37]. Response shift may occur because of a change in the respondent's internal standards of measurement (scale recalibration) [38], conceptualization of the health condition (concept redefinition) [39], or values [40].

Another explanation for disagreement between direct and indirect utilities might reside in the selection of specific functional domains within HUI systems and the way the domains are combined to generate a multi-attribute utility function. In the HUI systems, similar to many generic questionnaires designed to evaluate quality of life, no disease label is attached and only few aspects that determine quality of life of an individual are captured and summarized as a global score. In VAS and SG valuation methods, however, the individual evaluates his or her own health state based on a holistic concept and determines a global value for a global notion that includes not only his or her level of functioning but also the diagnosis, probable outcomes, and available treatment options. In addition to this, one individual might value a domain, such as mobility, twice as much as a different domain, such as cognition. Another person might value it only half as much. In indirect measures, the multi-attribute utility function gives a single global assessment score for the HRQL, thereby suppressing the interpersonal heterogeneity in preferences for domains. Direct measures, however, reflect this heterogeneity [41,42]. Some studies have found that, for the majority of individuals, incorporating the relative importance of domains in indirect HRQL measurement has little effect on the accuracy of utility estimation [43]. While this means that consideration of relative domain preferences does not significantly change the results at the group level, as the authors confirmed, it might be important at the individual level of analysis.

Another source of disagreement could stem from the method we used to obtain SG "utilities" from VAS "values". VAS and SG techniques both quantify preferences; however, since their measurement approach is different, there is an essential dissimilarity between their scores. In health status assessment, the subject is asked to compare two or more health states and then make a choice between them or scale the alternatives. In the VAS technique, the question is framed under certainty, thus VAS is regarded as a measurable value function and represents the strength of preference under certainty. In contrast, in the SG technique, which is based on the expected utility theory axioms [9,44-46], the question is framed under uncertainty, thus SG is considered as a utility function and represents the strength of preference under uncertainty [16]. As a result, SG "utilities" convey some extra information about the subject's risk attitude which is not included in VAS "values". Dyer and Sarin [47] named this extra information as "relative risk attitude" which is different from the conventional concept of risk attitude. These authors explained that as the quantity of risky alternatives is increased or decreased, the marginal value of additional units of those risky alternatives might change and that this change in marginal value should be separated from people's attitude toward risk. They suggested that an individual's relative risk attitude might be independent of the attribute on which his or her preferences are assessed and consequently proposed that it might be appropriate to obtain "values" and then transform them to "utilities" using a relative risk attitude obtained from others who represent the decision maker [47]. Based on the consistent observation that VAS values are lower than SG utilities, and that both scores are anchored at dead = 0.00 and healthy = 1.00, Torrance and colleagues concluded that if there is a systematic relationship between the two measures, it should be a concave curve that passes through 0 and 1 [16]. They determined that a power conversion function fulfils these criteria.

In order to test whether the effect of power conversion might help explain the lack of perfect agreement between direct and indirect utility measurements, we also assessed the agreement between VAS and HUI scores and compared them with ICCs between SG and HUI scores (results not shown). In all three assessments (A, B and C) and for both HUI2 and HUI3, transformation of VAS values to SG utilities decreased the agreement. Better agreement between rating scales and HUI scores than between SG and HUI scores has also been noted by Bosch et al. [48] in a study conducted on patients with intermittent claudication. These results support the claim that power conversion might not be the best function to transform VAS values to SG utilities. Other studies have examined the relationship between values and utilities and were unable to confirm the power function with their data [49]. However, even though the appropriateness of using power conversion to transform VAS values to utility scores is uncertain, we believe this factor has not significantly contributed to the observed disagreement. We calculated Pearson coefficients as well as ICCs in our analysis (Table 3). Pearson coefficient only examines how well the ranking of health states from the best to the worst are comparable between SG and HUI. In the ICC method on the other hand, the absolute values of utilities are taken into account. Therefore it is reasonable to expect that Pearson coefficients will be greater than ICC values. Comparison of the Pearson correlation coefficients and ICCs showed that in almost all assessments, the Pearson coefficient was greater than the corresponding ICC. However, the magnitudes of the differences were negligible (maximum 7%) and none of them were statistically significant. Therefore we expect factors, other than power conversion, to be responsible for the detected disagreement. It is worth reminding that in development of the HUI2 and HUI3 systems, the same method (power conversion) was used to estimate SG utilities [3,4], therefore whatever the effect of power conversion is, it is common between the SG utilities calculated in this study and HUI scores obtained from scoring formulas in our study. However, our results were consistent across several power functions (Table 3). Interestingly, the smallest ICC was consistently obtained using the same power function as has been used to generate the HUI2.

### Agreement between direct and indirect utilities at the group level

At the group level, direct and indirect utilities showed important and statistically significant differences. However, after observing strong agreement at the individual level, we expected otherwise. This is because direct measures preserve individual variability in utility scores, whereas in the scoring formulas of HUI systems, individual utilities are averaged and this variability is suppressed. One explanation for disagreement at the group level is the concept of response shift, as discussed above. If we agree that chronically ill patients usually become accustomed to their situation, patient and community utilities should not match and patient utilities should exceed those of the community. This argument is supported by our findings because, regardless of the effect of adjustment, the observed differences in our t-test analysis are consistently positive in all eight power functions and three assessments.

Although our analysis demonstrated obvious differences between the two HUI systems, we did not intend to compare HUI2 and HUI3 systems in this study. Similar relationship between HUI2 and HUI3 scores has been reported and possible explanations for such differences have been presented elsewhere [4,25,27,28].

### Study limitations

In measuring preferences for health states, a predefined hypothetical health state can be explained to the respondent. Alternatively, the subject can be asked to evaluate his or her own SDCS [2]. In this study, VAS scores were obtained from patients with their SDCS in mind. If we assume that a respondent's conceptualization of health status included some other dimensions not included in the HUI2 and HUI3 systems, then in this study we have actually compared different health states to each other. This limitation might explain at least some part of the observed disagreement between direct and indirect utilities.

A power conversion specific to this study was not estimated. It seems that individuals do not have a context-independent relative risk attitude and a single power conversion can not be found to convert VAS scores to SG scores [15]. Torrance et al. explained that although context biases have been identified in several studies, the relationship between VAS scores and SG utilities can be modelled by a power curve specific to the study [16]. They emphasize that the power function should be developed within the same study. In development of the HUI2 and HUI3 systems, VAS scores and SG utilities were measured for a limited number of health states in the same study to estimate the power function which was used to transform the scores. However, there are other studies that have not estimated their power function within the context of that study and applied a power function reported by others [11,12]. Although this limitation could have affected the results of current study, several power conversions were examined to minimize this shortcoming and the results were robust to utilization of various power functions.

VAS measurements have several problems. First, if the top and bottom anchors of VAS are not clearly defined (e.g. dead), comparison of scores between individuals might be invalid. The anchors for the VAS used in this study (as included in the EQ5D questionnaire) were labeled "best imaginable health state" and "worst imaginable health state". Clearly, these anchors can be conceptualized by individuals differently. However, on the VAS used to develop the HUI systems, the anchors were also labeled "best desirable" and "worst desirable" and were not clearly defined. Furthermore, VAS measurements are prone to several measurement biases such as spacing-out bias, end-aversion bias, and context biases [13,15]. In this study, the effect of end-aversion bias at the upper end of the scale has been adjusted. However, there are other types of adjustment that could have been used to improve the results, such as Parducci and Wedell's range-frequency model [50].

## Conclusion

National guidelines in Canada and the United States have recommended using community-preference-based valuation methods, such as the HUI systems, for economic evaluations and HRQL assessments [18,19]. Due to the simplicity of VAS measurements for both respondents and researchers, there might be a tendency to measure patient preferences using a VAS, adjust for biases, and then convert the scores to utilities using a power transformation function. Our study showed that for group level analysis, VAS-derived utility scores are not good substitutes for HUI scores.

Furthermore, our results support the existence of response shift phenomenon in chronically ill patients, explaining why patients usually give higher utility scores to their condition compared to the general public. This might increase the incremental cost-effectiveness ratio for some preventive health interventions performed from the patient's perspective compared to community's perspective. Consequently, resource allocation decisions and the selection of health interventions for funding might greatly depend on the source of preferences or on the assessment technique.

More research is needed to assess the agreement between direct and indirect preference measurement methods at the individual and group levels.

## Authors' contributions

AAR participated in the design of the study, performed the background research, carried out the data analysis and interpretation, and wrote the manuscript. AHA participated in the design of the study and supervised the research activities. CAM participated in the design of the study, statistical analysis, interpretation of the results, and writing the manuscript. All authors read and approved the final manuscript.
